# Reporting of Positive Results in Randomized Controlled Trials of Mindfulness-Based Mental Health Interventions

**DOI:** 10.1371/journal.pone.0153220

**Published:** 2016-04-08

**Authors:** Stephanie Coronado-Montoya, Alexander W. Levis, Linda Kwakkenbos, Russell J. Steele, Erick H. Turner, Brett D. Thombs

**Affiliations:** 1 Lady Davis Institute for Medical Research, Jewish General Hospital, Montréal, Québec, Canada; 2 Department of Psychiatry, McGill University, Montréal, Québec, Canada; 3 Department of Mathematics and Statistics, McGill University, Montréal, Québec, Canada; 4 Department of Psychiatry, Oregon Health & Science University, Portland, Oregon, United States of America; 5 Department of Psychiatry, Portland Veterans Affairs Medical Center, Portland, Oregon, United States of America; 6 Department of Epidemiology, Biostatistics, and Occupational Health, McGill University, Montréal, Québec, Canada; 7 Department of Medicine, McGill University, Montréal, Québec, Canada; 8 Department of Educational and Counselling Psychology, McGill University, Montréal, Québec, Canada; 9 Department of Psychology, McGill University, Montréal, Québec, Canada; 10 School of Nursing, McGill University, Montréal, Québec, Canada; Stanford University, UNITED STATES

## Abstract

**Background:**

A large proportion of mindfulness-based therapy trials report statistically significant results, even in the context of very low statistical power. The objective of the present study was to characterize the reporting of “positive” results in randomized controlled trials of mindfulness-based therapy. We also assessed mindfulness-based therapy trial registrations for indications of possible reporting bias and reviewed recent systematic reviews and meta-analyses to determine whether reporting biases were identified.

**Methods:**

CINAHL, Cochrane CENTRAL, EMBASE, ISI, MEDLINE, PsycInfo, and SCOPUS databases were searched for randomized controlled trials of mindfulness-based therapy. The number of positive trials was described and compared to the number that might be expected if mindfulness-based therapy were similarly effective compared to individual therapy for depression. Trial registries were searched for mindfulness-based therapy registrations. CINAHL, Cochrane CENTRAL, EMBASE, ISI, MEDLINE, PsycInfo, and SCOPUS were also searched for mindfulness-based therapy systematic reviews and meta-analyses.

**Results:**

108 (87%) of 124 published trials reported ≥1 positive outcome in the abstract, and 109 (88%) concluded that mindfulness-based therapy was effective, 1.6 times greater than the expected number of positive trials based on effect size d = 0.55 (expected number positive trials = 65.7). Of 21 trial registrations, 13 (62%) remained unpublished 30 months post-trial completion. No trial registrations adequately specified a single primary outcome measure with time of assessment. None of 36 systematic reviews and meta-analyses concluded that effect estimates were overestimated due to reporting biases.

**Conclusions:**

The proportion of mindfulness-based therapy trials with statistically significant results may overstate what would occur in practice.

## Introduction

Mindfulness-based therapies (MBT), which include mindfulness-based stress reduction (MBSR) programs and mindfulness-based cognitive therapy (MBCT), have been described as feasibly delivered, low-cost, evidence-based options for managing stress, reducing mental health symptoms, and preventing relapse of depression [1–4]. MBSR is an 8-week group-based program, designed to reduce stress through mindful awareness [5, 6]. The program consists of weekly 2 to 2.5 hour sessions, a whole-day retreat, and independent daily meditation and yoga. MBCT additionally incorporates cognitive therapy into the sessions [5, 7]. MBSR can be led by trained para-professionals or by laypersons [8], whereas MBCT must be led by a licensed health care provider [9].

MBSR and MBCT have been reported to improve mental health outcomes among patients with psychiatric conditions (e.g., depression [1, 10], anxiety [11, 12], posttraumatic stress disorder [13], eating disorders [14], substance use disorders [15]), and other medical conditions (e.g., diabetes [16], hypertension [17], cancer [18], arthritis [19], obesity [20], heart disease [21], stroke [22]). In the United Kingdom, MBCT has been recommended by the National Institute for Health and Care Excellence to prevent depression relapse [23].

A concern, however, is that the overwhelmingly statistically significant results in favor of MBSR and MBCT interventions that can be seen in the published literature, despite very low power in many studies, may be influenced by reporting biases. Reporting biases are said to occur when statistically significant or “positive” outcomes have been preferentially published compared to non-significant or “negative” outcomes [24–26]. Reporting biases include (1) study publication bias, in which positive studies tend to be published, whereas negative studies are not; (2) selective outcome reporting bias, in which outcomes published are chosen based on statistical significance with non-significant outcomes not published; (3) selective analysis reporting bias, in which data are analyzed with multiple methods but are reported only for those that produce positive results; and (4) other biases, such as relegation of non-significant primary outcomes to secondary status when results are published [24–28].

Meta-analyses of MBT have either not assessed reporting biases [29–32] or have attempted to assess the possibility of publication bias and reported that it was not present or not likely to have influenced findings [33–42]. Studies that have attempted to detect publication bias have used statistical or graphical methods, such as visual examination or statistical tests for asymmetry of funnel plots or procedures that aim to identify and correct for funnel plot asymmetry, such as trim and fill [43–46]. These methods assess whether larger effect sizes are associated with smaller trials among published trials, which would suggest that relatively small, non-significant trials may tend to go unpublished. These tests are commonly used, but they are not likely to detect reporting biases, if present, when there are fewer than 10 to 20 included trials and may require very large numbers of trials in some circumstances. They are also not appropriate when most studies have limited sample sizes, or when there is relatively little variance in sample sizes [45, 46], all of which are common in MBT trials.

Some studies of MBT [34, 37, 39, 41, 42] have also used the fail-safe N method, which attempts to determine the number of additional trials with zero intervention effect that would be needed to increase the overall P value to above 0.05. This method is generally discouraged, however, due to methodological concerns and because it emphasizes statistical significance or non-significance rather than the magnitude of an estimated intervention effect and associated confidence intervals [47].

The authors of a recent meta-analysis of 47 trials of MBT for psychological stress and well-being [29] concluded that they could not conduct quantitative tests of publication bias due to the relatively small number of trials reporting most of the outcomes they evaluated. Instead, they reviewed trial registries and found 5 trials that were completed at least 3 years before their review, but did not publish all registered outcomes, and 9 completed trials for which an associated publication was not found, suggesting that reporting biases are sometimes present even if not easily detected using standard methods [29].

We have observed anecdotally that there seem to be few examples of published MBT trials without statistically significant results, even though many existing trials appear to have been conducted with very low statistical power. Thus, our objectives were to (1) characterize the degree to which published MBT trials report statistically significant results in favor of MBT interventions; (2) attempt to evaluate the plausibility of the number of statistically significant results; (3) evaluate MBT trial registrations and subsequent publication status to assess the potential influence of study publication bias and selective outcome reporting bias on the number of positive trials; and (4) evaluate systematic reviews and meta-analyses on MBT to determine whether reporting bias has been assessed and, if so, what conclusions have been drawn.

## Methods

### MBT Trials

#### Search Strategy and Identification of Eligible RCTs

The CINAHL, Cochrane CENTRAL, EMBASE, ISI, MEDLINE, PsycInfo, and SCOPUS databases were searched on July 4, 2013. See S1 Appendix for search terms.

Our main objective was to characterize the degree to which published MBT trials have reported statistically significant results, not comparative effectiveness. Thus, RCTs published in any language, including dissertations that appeared in indexed databases, were eligible if they evaluated the effect of MBT versus usual care, placebo, or other inactive controls (e.g., waitlist, sham control) on mental health outcomes in any population. MBT was defined as a group-based intervention in which standard MBT components comprised the core of the intervention [5, 7]. Shortened MBT interventions were included if they provided at least 4 sessions over 4 or more weeks and include core MBT elements (e.g., meditation, yoga). RCTs of interventions that included a substantive component not typically included in MBT and not available to the control group (e.g., exercise, art therapy, weight loss programs) were excluded. Meditation-based interventions not described as mindfulness-based and/or not including key components of MBSR (e.g., yoga) or MBCT (e.g., focus on cognitive distortions) were excluded. Internet-based interventions were also excluded. Because we sought trials of interventions intended to influence mental health, eligible RCTs had to report at least one outcome reflecting mental health status (e.g., symptoms of depression, anxiety) in the abstract.

Search results were downloaded into the citation management database RefWorks (RefWorks, RefWorks-COS, Bethesda, MD, USA) and duplicates were removed using the RefWorks duplication check and manual searching. Two investigators independently reviewed articles for eligibility. If either deemed an article potentially eligible based on title/abstract review, then a full-text review was completed. Disagreements after full-text review were resolved by consensus. Translation was done for non-English articles.

#### Data Extraction

Two investigators independently extracted and entered data items from eligible RCTs into a standardized spreadsheet; discrepancies were resolved by consensus. When there was more than one publication on the same RCT, we extracted data for the RCT as a unit, incorporating information from all publications together. Identification of multiple publications from the same RCT was done by cross-referencing authors and co-authors, patient characteristics, and countries. In cases where it was not clear whether publications reported data from the same RCT, we contacted study authors.

#### Identifying MBT Trials with “Positive” Results

For most included trials it was not possible to identify a pre-defined, single primary outcome variable, and, in many trials, it was not possible to even identify a single primary outcome, whether or not pre-defined. Most trial reports included multiple outcome variables with no indication of primacy or included multiple “primary” outcome variables with no statistical adjustment. Since we could not identify a single primary outcome variable in most cases, in order to attempt to determine if a trial had been reported as a positive trial, we used a classification method based on a method published by Kyzas et al. [48] as our primary classification method. RCTs were classified as negative if all between-groups mental health outcomes reported in the abstract were statistically non-significant or as positive if at least one was statistically significant. Since this method could over-identify trials as positive, we also classified studies as negative or positive based on published study conclusions. Conclusions of study abstracts were coded as unequivocally supporting the effectiveness of MBT (positive), suggesting that MBT was not effective (negative), or as “mixed or inconclusive.”

Based on our primary classification method, negative RCTs were further coded to indicate whether results were presented with a caveat, defined as a statement made by investigators to mitigate the lack of statistical significance [48, 49]. Caveats included describing non-significant results as representing “trends” for a therapeutic effect; suggestions that other, larger, or different studies would likely find a positive effect; or arguments that MBT was still important to provide for other reasons [48, 49]. For positive RCTs, we coded whether all results reported in the abstract were statistically significant or whether there was at least one non-significant result. If no between-groups results were reported in the abstract, we coded within-group pre-post results from the abstract to determine positive versus negative status.

The effectiveness of mental health therapies in trials may depend on whether they are delivered by highly trained professionals, as in MBCT, versus professionals with less clinical training, as in MBSR; whether the patient sample is a symptomatic clinical sample versus a non-clinical sample; and whether a minimum symptom threshold is required for enrollment [50–54]. Thus, in addition to reporting totals for all MBT trials, we also categorized trials into subgroups of (1) RCTs of MBCT versus other MBT interventions, either MBSR or a similar mindfulness meditation-based program; (2) Clinical versus non-clinical patient sample; and (3) Trials with a mental health symptom threshold requirement for patient eligibility versus no such requirement. Clinical samples were defined as including only patients with a defined mental health (e.g., depression) or medical (e.g., arthritis) condition. Non-clinical samples included general population, employee, or student samples, for instance.

#### Plausibility of Proportion of RCTs with Positive Results

We initially intended to evaluate the plausibility of the number of RCTs with positive results using the test for excess significance, which was developed by Ioannidis and Trikalinos [55, 56]. The test is based on the idea that all forms of reporting bias, including publication bias and selective reporting of outcomes or analyses, result in an exaggerated number of statistically significant results in published trial reports. Thus, the test for excess significance [55, 56] evaluates whether the number of observed positive trials exceeds the number expected based on the statistical power of published trials. It does not depend on the strong assumption that sample size is associated with reporting bias, as in graphical and regression-based methods and may be more robust than other tests in the context of small numbers of trials and limited variability in trial sample sizes [55, 56]. Pragmatically, the test for excess significance assesses whether the observed number of positive MBT RCTs is significantly larger than the expected number given a particular estimated effect size. The observed number is obtained by summing the number of positive studies, and the expected number is the sum of the power of all included RCTs based on the estimated effect size.

We encountered three substantive barriers, however, to applying the test for excess significance in this group of studies, as we had originally intended. First, we were not able to identify a single primary outcome variable in most studies on which to base a study-specific effect size estimate. Second, related to this, we did not believe that we could reasonably estimate an unbiased “true” effect size upon which to base an estimate of statistical power for all MBT trials, which is needed for the text of excess significance. In the context of substantial selective reporting, a meta-analysis based effect estimate would exaggerate actual effectiveness and underestimate excess statistical significance. Third, given the clinical heterogeneity of the included studies, it was reasonable to assume that there was substantial heterogeneity of effects across studies, and substantive heterogeneity can also lead to a greater number of statistically significant results, beyond reporting biases.

Thus, we did not conduct a statistical test for excess significance. Instead, we presented the overall number of positive trials and the number of positive trials in each subgroup. For comparison purposes, we also presented the number that would have been expected if the true effect size were the same as the effect size reported in a recent meta-analysis of trials of individual psychotherapy for adult depression, d = 0.55 [57]. We used this type of therapy as a reference point because it is another intervention that is intended for mental health symptoms and its therapeutic effects on depression have been well-studied, compared to most other mental health treatment conditions. Furthermore, we believe that d = 0.55 was a conservative estimate for our purpose, in that it is almost certainly an overestimate of the true effect of MBT therapies. This is because individual depression therapy is administered in an individual format, is provided by a trained mental health practitioner, and is delivered to patients with a defined clinical condition. These characteristics all tend to result in greater effects compared to therapies administered in group formats by non-mental health professionals to treatment recipients who may not have a defined mental health condition or exceed any symptom threshold for eligibility [50–54], as is the case in many MBT intervention trials. Furthermore, this effect estimate is substantially greater than all effect sizes reported in a recent Agency for Healthcare Research and Quality (AHRQ) systematic review that found that meditation programs, including MBT reduced symptoms of anxiety, depression, and pain by 0.22 to 0.44 standard deviations [29].

Power for each study that we included was calculated using the pwr package in R [58]. The expected number was calculated with the understanding that any differences between the observed number of MBT trials and the expected number based on this effect estimate could have occurred because the effect estimate was not accurate for MBT trials, because of effect heterogeneity, due to differences in study quality between the trials used to generate that effect and the trials in the present study, because of reporting biases in the MBT trials, or some combination thereof. We also calculated the effect size that would have been necessary for the expected number of positive studies to equal the observed number with the same understanding that any difference could have been due to multiple factors.

### MBT Trial Registrations

We examined MBT trial registrations to assess the degree to which publication bias and selective outcome reporting may have influenced the number of positive trials we encountered.

#### Search Strategy

We searched 3 trial registries: ClinicalTrials.gov, the Standard Randomized Controlled Trial Number Register, and the World Health Organization’s (WHO) International Clinical Trials Registry Platform, which is a central database that provides access to multiple national or region-specific registries (see S1 Appendix for search terms). We included all registrations of RCTs completed as of December 31, 2010, which compared MBT to an inactive comparator and that reported mental health outcomes, consistent with our eligibility criteria for published RCTs of MBT. We included trials completed by as of December 31, 2010 to allow at least 30 months between trial completion and our search for published results, consistent with the methods of a recent study on publication patterns following registration [59]. The completion date was defined by ClinicalTrials.gov as “final data collection date for primary outcome measure.” For trial registrations in registries other than ClinicalTrials.gov that did not provide a date for completion of data collection, we contacted investigators directly. Studies with unknown status were considered completed if 2 or more years had lapsed since the last trial registry update and if the expected study completion date was December 31, 2010 or earlier. Trials listed as terminated or withdrawn were considered completed and were included.

Results were downloaded into an Excel database. Duplicate registrations were identified in the WHO registry platform automatically, and any additional duplicates across registries were identified by manual search. Two investigators independently reviewed trial registrations for eligibility with any disagreements resolved by consensus.

#### Publication Status of Registered MBT Trials

Two investigators independently reviewed each trial registration for listed publications of trial results in peer-reviewed journals. If none were listed, search results from the electronic database search were reviewed for published RCTs (see above section regarding eligible RCTs) to attempt to identify published results. If none were found, MEDLINE and PsycInfo were additionally searched for results published in peer-reviewed journals using the trial registration number and, if unsuccessful, using the intervention, condition studied, and the name of the listed principal investigator (e.g., Bremner AND mindful*). Each trial registration was classified as having published or not published trial results in a peer-reviewed journal or indexed doctoral dissertation within 30 months of completion. For trials published online ahead of print, the date when the trial was made available electronically was used as the publication date [59].

#### Risk of Selective Outcome Reporting in Registered MBT Trials

The risk of selective outcome reporting increases when, prior to data collection, there is no clear declaration of a single primary trial outcome or, in the case of multiple primary trial outcomes, when there is no clear declaration of those outcomes with a plan to adjust for multiple analyses [24, 60–62]. Using a method similar to that described by Mathieu et al. and used subsequently by others [60–62], “adequately registered” MBT trials were defined as trials that registered a single primary outcome (or multiple primary outcomes with appropriate adjustment), specified the primary outcome measure, the time point when it would be assessed, and the metric (e.g., continuous, dichotomous with specified cutoff threshold). As a sensitivity analysis, we reclassified trial registration adequacy without requiring specification of the metric. Registration adequacy was assessed by two investigators independently with any disagreement resolved by consensus.

### Assessment of Possible Reporting Biases in Systematic Reviews and Meta-Analyses of MBT

#### Search Strategy and Identification of Eligible Systematic Reviews and Meta-Analyses

The CINAHL, Cochrane CENTRAL, EMBASE, ISI, MEDLINE, PsycInfo, and SCOPUS databases were searched on August 26, 2013 for recent systematic reviews and meta-analyses, published since January 1, 2011, on the effectiveness of MBT for improving mental health outcomes (see S1 Appendix for search terms). We restricted the search to this approximately 3-year period to obtain recent systematic reviews and meta-analyses that reflect relatively current practices.

Systematic reviews and meta-analyses published in any language were eligible if they reviewed the effectiveness of MBT on mental health outcomes. The same review procedure was used as for individual published RCTs of MBT, described above.

#### Data extraction

Two investigators independently extracted and entered data items into a standardized spreadsheet, with discrepancies resolved by consensus. For each systematic review or meta-analysis, they determined whether authors conducted a statistical test (e.g., asymmetry test, fail-safe N, regression analysis) or a visual inspection of funnel plots to assess possible reporting bias. They also reviewed the abstract and discussion section of each systematic review or meta-analysis to determine whether authors mentioned the possibility that reporting biases could have influenced results.

## Results

### Statistical Significance in Results of Published MBT RCTs

#### Search results

**Fig 1:**
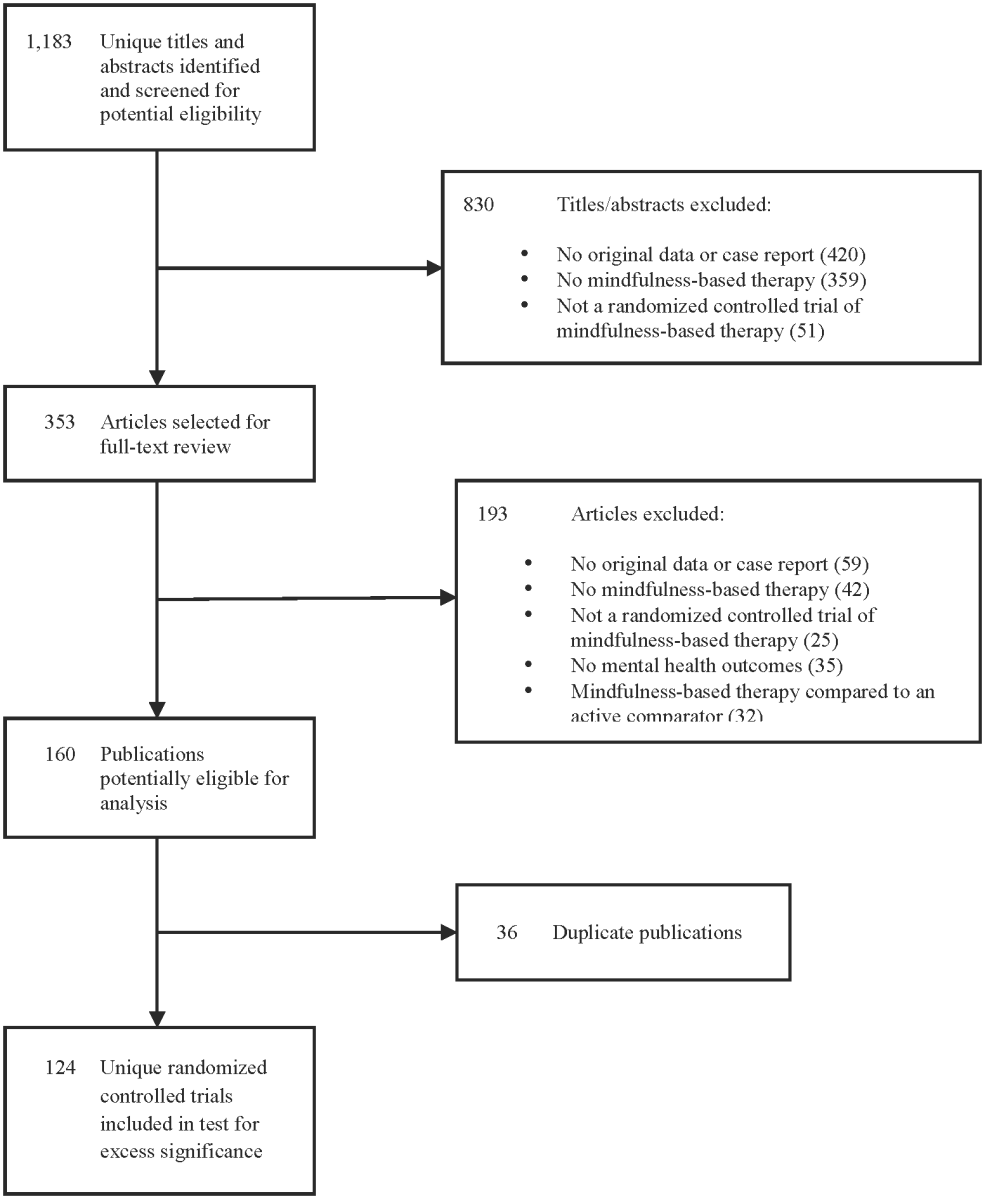
PRISMA Flow Diagram of Selection of Published Randomized Controlled Trials of Mindfulness-based Therapy. PRISMA flow diagram of selection of published randomized controlled trials of mindfulness-based therapies on mental health outcomes, including reasons for and number of excluded trials.

The electronic database search yielded 1,183 unique publications for review, of which 830 were excluded after review of titles and abstracts and 193 after full-text review. There were 36 related publications that reported on the same RCT in multiple publications, leaving 124 unique MBT RCTs (see Fig 1).

#### Characteristics of Published MBT RCTs

Of the 124 included RCTs, there were 62 RCTs (50%) from North America, 42 (34%) from Europe, and 20 (16%) others. There were 4 RCTs (3%) published before 2000, 40 (32%) between 2000 and 2009, and 80 (65%) in 2010 or later. The total number of patients analyzed in the combined intervention and control groups ranged from 10 to 357 participants per RCT. There were 58 trials (47%) with 10–49 total participants analyzed, 40 (32%) with 50–99, and 26 (21%) with 100–357. The mean number of patients analyzed in each trial was 70.3, and the median was 54.5. There were 13 trials that were published only as dissertations and retrieved via electronic databases, but were not published in a peer-reviewed journal.

According to the three key subgroup classifications, there were 28 RCTs (23%) of MBCT versus 96 (77%) of other MBTs; 41 RCTs (33%) that required a minimum symptom threshold for trial eligibility versus 83 (67%) that did not; and 83 RCTs (67%) with clinical samples versus 41 (33%) with non-clinical samples. Of the 83 RCTs with clinical populations, there were 36 with psychiatric patients, 12 with chronic pain patients, 11 with cancer patients, 7 with obese or diabetic patients, and 17 with patients with other conditions. See S2 Appendix for characteristics of all included RCTs, including subgroup classifications.

Of the 124 RCTs, 26 (21%) had a registration record, including 21 (17%) that were registered prior to data collection. Of these, 12 were listed as completed by December 31, 2010 and included in our analysis of trial registrations and publication status (see section below, Evaluation of MBT Registrations); 1 was registered in the Centre for Clinical Trials registry (http://www.cct.cuhk.edu.hk/cctwebsite/default.aspx), which was not one of the registries that we searched, so it was not included in our trial registry analysis; and 8 were registered prospectively, but the completion date was after 2010, which was an exclusion criteria for trial registry analysis.

#### Positive Results in RCTs

Of the 124 included RCTs, 108 (87%) were classified as positive and 16 (13%) as negative based on reporting at least one positive outcome in the abstract. When classifications were instead based on study conclusions, there were 109 (88%) clearly positive studies that concluded that MBT was effective, 11 (9%) with mixed conclusions, and 4 (3%) negative studies that concluded that MBT had not been effective. There was a 91% rate of agreement between the two methods (113 of 124 trials). Of the 13 RCTs published only as dissertations, 8 (62%) were classified as positive based on both methods.

Of the 108 positive RCTs based on our primary classification method, 94 reported at least one significant between-groups mental health outcome, and 14 did not report any between-groups mental health outcomes, but reported at least one significant within-group mental health outcome. In the abstracts of the 16 negative RCTs, 11 reported positive pre-post changes for the MBT group in addition to negative between-groups results. Additionally, 5 of the 16 negative RCTs included a caveat. Only 3 reported negative between-groups results without highlighting significant within-group findings or providing a caveat.

For an assumed effect size of d = 0.55, the expected number of positive RCTs was 65.7. As shown in Table 1, the overall ratio of observed-to-expected positive studies was 1.6 times. Within the subset of 15 studies with power <0.25, the observed-to-expected ratio was 3.6 times; among 45 studies with power between 0.25 and 0.50, the observed-to-expected ratio was 2.5 times. As shown in Table 2, results for the observed to expected ratio were similar and consistent across subgroups. See S3 Appendix for power value used in calculations for individual studies.

**Table 1:** Summary of Observed and Expected number of Positive Studies with Power Calculation Based on Effect Size d = 0.55^a^.

Statistical Power (1 − β)	Total Number of RCTs	Total Number of Patients Analyzed in RCTs^b^	Observed Number (%) of Positive Studies	Expected Number (%) of Positive Studies^c^	Ratio of Observed Number / Expected Number of Positive Studies
0 ≤ (1 − β) < 0.25	15	10–29	10 (67%)	2.8 (19%)	3.6
0.25 ≤ (1 − β) < 0.50	45	24–51	39 (87%)	15.7 (35%)	2.5
0.50 ≤ (1 − β) < 0.75	34	54–92	33 (97%)	21.1 (62%)	1.6
0.75 ≤ (1 − β) ≤ 1.00	30	95–357	26 (87%)	26.1 (87%)	1.0
Total	124	10–357	108 (87%)	65.7 (53%)	1.6

Abbreviation: RCTs = Randomized controlled trials

^a^ See S3 Appendix for power values for individual studies.

^b^ Statistical power is influenced by the distribution of patients between treatment and control groups. Thus, total sample size is not directly indicative of statistical power.

^c^ Expected number of positive studies calculated by summing the power for all studies.

**Table 2:** Summary of Observed and Expected number of Positive Studies for Key Subgroups with Power Calculation Based on Effect Size d = 0.55^a^.

Key Subgroups	Total Number of RCTs	Total Number of Patients Analyzed in RCTs	Observed Number (%) of Positive Studies	Expected Number (%) of Positive Studies^b^	Ratio of Number Observed / Expected Number of Positive Studies
**Type of Therapy**					
MBCT	28	16–205	26.0 (93%)	14.7 (53%)	1.8
Other MBT	96	10–357	82.0 (85%)	51.1 (53%)	1.6
**Sample Used**					
Clinical	83	10–282	72.0 (87%)	46.7 (56%)	1.5
Non-clinical	41	17–357	36.0 (88%)	19.0 (46%)	1.9
**Threshold Requirement**					
Yes	41	10–205	39.0 (95%)	22.3 (54%)	1.7
No	83	12–357	69.0 (83%)	43.5 (52%)	1.6

Abbreviations: RCTs = Randomized controlled trials; MBCT = Mindfulness-based cognitive therapy; MBT = Mindfulness-based therapy

^a^ See S3 Appendix for power values for individual studies.

^b^ Expected number of positive studies calculated by summing the power for all studies.

To obtain an expected number of positive studies of 108, which was the number of observed positive studies, the true effect size would have needed to be d = 1.03.

### Evaluation of MBT Trial Registrations

#### Search results

**Fig 2:**
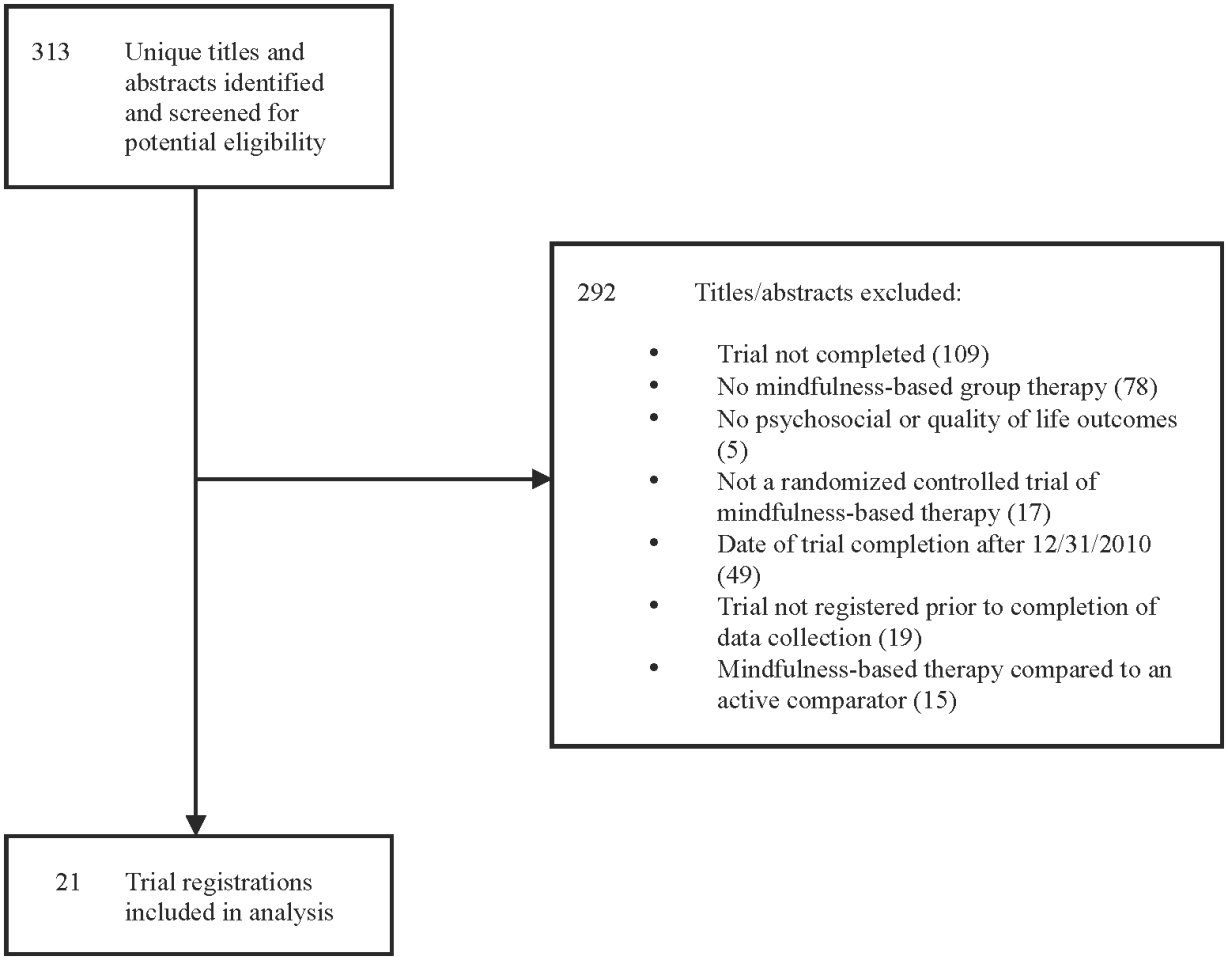
PRISMA Flow Diagram of Selection of Trial Registrations of Completed Randomized Controlled Trials of Mindfulness-based Therapy. PRISMA flow diagram of trial registrations of completed randomized controlled trials of mindfulness-based therapy on mental health outcomes, including reasons for and number of excluded trial registrations.

The trial registry search yielded 313 unique registrations, of which 292 were excluded, leaving 21 eligible trial registrations of MBT RCTs (see Fig 2).

#### Characteristics of Trial Registrations of MBT RCTs

Of the 21 registered trials, 8 (38%) were published within 30 months of trial completion. All 8 reported positive outcomes in the published abstract and were classified as positive studies based on their conclusions.

None of the 21 registered trials adequately specified a single primary outcome, including the outcome measure, the assessment time, and the metric. When metric specification was not required, there were 2 (10%) adequate trial registrations and 19 trials (90%) not adequately registered. These 19 registrations were classified as inadequate because multiple outcomes were listed without specifying a primary outcome or plan to adjust statistically for multiple outcomes (n = 16), because a specific measure was not listed for the primary outcome (n = 2), or because a time point was not specified for the primary outcome (n = 1). See S4 Appendix for details.

### Assessment of Possible Reporting Biases in Systematic Reviews and Meta-Analyses of MBT

#### Search results

**Fig 3:**
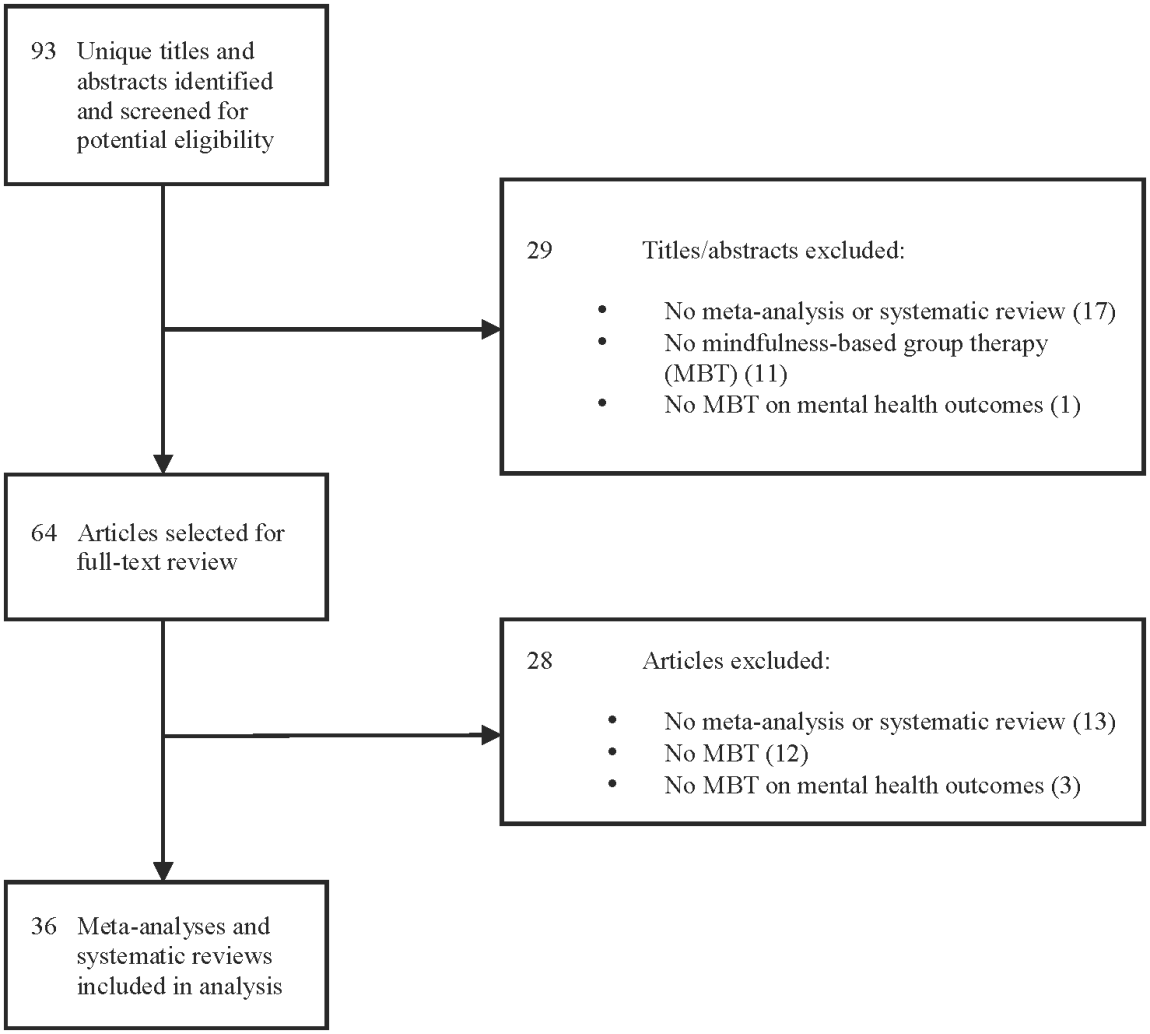
PRISMA Flow Diagram of Meta-Analysis and Systematic Review Selection Process for Study. PRISMA flow diagram of recent meta-analyses and systematic reviews of mindfulness-based therapy on mental health outcomes, including reasons for and number of excluded reviews.

The search for systematic reviews and meta-analyses yielded 93 unique articles for review, of which 29 were excluded after review of titles and abstracts and 28 after full-text review, leaving 36 systematic reviews and meta-analyses eligible for evaluation (see Fig 3).

#### Characteristics of Systematic Reviews and Meta-Analyses of MBT

As shown in S5 Appendix, only 2 of the 36 systematic reviews and meta-analyses included >20 RCTs. Of the 36, 14 (39%) conducted a statistical or visual test to assess possible reporting bias. Of these, 9 reviews concluded that there was no apparent bias, 4 were inconclusive or stated that presence of publication bias was possible, and 1 that publication bias was present, but in the opposite direction (small effect sizes more likely to be published). None mentioned possible reporting bias in the review abstract.

## Discussion

The main finding of this study was that of 124 MBT RCTs that were reviewed, almost 90% were presented as positive studies when published. Furthermore, there were only 3 trials that were presented unequivocally as negative trials without alternative interpretations or caveats to mitigate the negative results and suggest that the treatment might still be an effective treatment.

For a point of reference, we compared the number of positive trials that we found to the number that would have been generated by a group of heterogeneous studies with a true effect size of d = 0.55, which is the effect size obtained from a recent meta-analysis of individual therapy for depression [57]. This effect likely overstates the actual effect size of MBT since MBT is often administered in groups by people who do not necessarily have professional mental health training to treatment recipients without defined diagnoses or levels of symptoms, all of which likely reduce effect sizes [50–54]. Furthermore, this effect estimate may be exaggerated even for depression treatments. A recent study of US National Institute of Health grants for psychological treatments for patients with depressive disorders found that the effect size of g = 0.52 among published studies was reduced to g = 0.39 when data for non-published studies was integrated [63]. Additionally, it is of note that d = 0.55 substantially exceeds effect estimates published in a recent AHRQ meta-analysis of meditative therapies [29]. Based on this reference point, there were 1.6 times as many positive MBT RCTs among the 124 RCTs we reviewed as would be expected if the true effect size of d = 0.55 in a relatively homogeneous group of trials. For trials with low power, this ratio was substantially higher. When we examined subgroups of only studies of MBCT (versus other MBTs), only studies with clinical populations (versus general population, employees, or students), and only studies that required mental health symptoms for enrollment, results were consistent.

Although there is reason to believe that the effect estimate we used as a reference point may have been too large and, thus, overestimated the expected number of positive studies, we cannot rule out several different explanations for why we found so many positive trials. One explanation is simply that we cannot be sure that the effect size that we used as a reference point was indeed an accurate estimate or that it overstated likely effectiveness, as we believe. Second, it may be the case that heterogeneity in study effects could have contributed to the high number of positive studies. Finally, it may be the case that reporting biases played an important role in this. This idea is supported by the fact that the tendency to generate more positive studies that would be expected was concentrated in smaller studies, although it is also possible that lower quality in smaller studies could have played a role.

Our review of trial registration records also suggest the possibility that reporting biases may have been an important factor. Of the 124 RCTs reviewed, only 21 (17%) were registered prior to data collection, even though 80 of the eligible RCTs were published recently (since 2010). When we examined trial registries, we identified 21 registrations of MBT trials listed as completed by 2010 and found that 13 (62%) remained unpublished 30 months after completion; of the published trials, all conveyed a positive conclusion. None of the 21 registrations, however, adequately specified a single primary outcome (or multiple primary outcomes with an appropriate plan for statistical adjustment) and specified the outcome measure, the time of assessment, and the metric (e.g., continuous, dichotomous). When we removed the metric requirement, only 2 (10%) registrations were classified as adequate. We evaluated more than 30 published systematic reviews and meta-analyses of MBTs, and none concluded that reporting biases likely exaggerated estimates of effect (see S6 Appendix). The authors of one recent meta-analysis, which was published after our search, on the other hand, raised concern about possible publication bias and other reporting biases based on trial registry records [29].

Ross et al. [59] recently reviewed publication patterns of all clinical trials funded by the United States National Institutes of Health, including pharmacological and non-pharmacological trials, and found that 46% of trials were published in a peer-reviewed journal within 30 months of the completion date documented in the trial registration. Overall, trials were followed for a median of 51 months post-completion, and 68% of trials were published in a peer-reviewed journal by the end of the study. The rate of publication of registered MBT trials within 30 months of completion in the present study was slightly lower (38%). It is possible, as reported by Ross et al., that additional trials will eventually be published, but this suggests that publication bias likely contributed to the findings of excess significance in the present study.

Thus, it may be the case that selective outcome reporting, as well as “data dredging” [56] and selective reporting of analyses may play important roles in the proportion of positive studies that we found among MBT RCTs in the present study. If one assumes that there is some effect of MBT on mental health outcomes, albeit a smaller effect than reported in published studies, the ability to selectively publish from multiple outcome options or multiple analyses could easily lead to exaggerated effect estimates and a rate of positive trial reports that exceeds plausibility, as we found in our study. Indeed, others have suggested that exaggerated effect sizes are problematic in trials that work with “soft” outcomes, as is typically the case in psychological or behavioral research [64–66], and that selective reporting of only some outcomes and analytical flexibility may be even larger problems than classic publication bias in psychological studies compared to “harder” sciences [64, 65].

Mathieu et al. [60] investigated trial registrations and outcomes from trials published in high-impact general and specialty medicine journals in 2008. Of the trials they reviewed, there were 186 that had been registered a priori, of which 147 (79%) were adequately registered with a clear description of a single primary outcome measure. On the other hand, a study of RCTs published in four top behavioral health journals between 2008 and 2009 found that only 1 of 63 RCTs was adequately registered, and within that one RCT, registered and published outcomes were discrepant [24]. Two recent studies of published trials in psychology and behavioral health journals reported similar results [61, 62]. The results of the present study suggest that inadequate trial registration and the lack of pre-specified primary trial outcomes may continue to plague research of non-pharmacological mental health interventions. The burden of registering a trial does not add substantively to the overall burden of designing, funding, conducting, and reporting a trial, and there are no real barriers to doing this. Thus, a greater focus on training of trialists is needed, as well as increasing rigor from journal reviewers and editors to ensure that only trials that are registered with enough information to compare pre-specified outcomes to reported outcomes should be published.

The very small number of trials that clearly declared negative results in the present study without caveats or “spin” also reminds us that when negative results are reported, they are often “spun” so that they appear to be equivocal or even positive findings [67]. One might reasonably expect that in the text of articles authors may attempt to justify their trials with caveats and discuss why statistically significant results were not found. However, the failure to provide a clear statement of non-significance in the abstract may serve to distort understandings of results, since many readers base their assessment of trial results on what is reported in the abstract.

In the present study, we found that most existing evidence syntheses either did not evaluate reporting biases or concluded that they were not present. The majority of these systematic reviews, which focused on a wide range of applications of MBT, included very small numbers of RCTs, which did not permit a statistical assessment of reporting biases. However, that would not have precluded approaches such as reviewing trial registries, in order to better understand the likelihood that completed trials of MBT may go unreported or that outcomes in published trials may be selectively reported. A meta-analysis, which was published subsequent to our search period and not included in our analysis, for instance, did not assess publication or other forms of reporting bias with statistical methods, but did identify patterns of non-publication and likely selective outcome reporting by reviewing MBT trial registrations [29]. This is an approach that can be utilized, whether or not statistical approaches are feasible.

There are a number of limitations that should be considered in interpreting the results of the present study. First, we were not able to conduct a statistical test to determine if there was excess significance bias. The results that we presented in comparison to a reference point effect size cannot rule determine the relative contributions to the relatively high number of positive results to the use of an inaccurate effect estimate as a reference point, to heterogeneity across studies, or to reporting biases. Generally, risk of reporting bias will be higher in fields with small, underpowered trials [68]; when there is a strong incentive for reporting positive results, which is often the case when professionals who practice a given psychological treatment also test it [69, 70]; and when there is prior documentation of bias in the field [68], as is the case with psychological treatments dealing with “soft” outcomes [64–66]. In the present study, only 30 of 124 trials (24%) had power of 75% or more when an effect size of d = 0.55 was assumed. Furthermore, there was evidence from trial registries that reporting biases may be problematic. Among registered, completed MBT trials that we reviewed, the majority were not published within 3 years of completion. Although it is not necessarily the case that unpublished studies were negative studies, it has long been established that negative studies are more likely to remain unpublished than positive studies [26]. Additionally, virtually no MBT registrations defined outcome variables with sufficient precision to compare to subsequently published trial results, which would reduce the likelihood of selective outcome reporting.

In summary, MBT appears to be a low-cost and easily implemented treatment that may be useful for providing effective mental health care to the large number of patients who are currently under-served [71]. However, the proportion of positive trials that are reported, despite small sample sizes and low statistical power are concerning. Although we could not determine with certainty the degree that reporting biases play a definitive role in this, there was evidence that this may be driving force. Investigators who conduct trials of MBT and other non-pharmaceutical interventions to improve mental health should register their trials with enough information so that readers can verify whether published outcomes match the pre-specified outcomes. In addition, journal editors and reviewers should routinely compare a priori defined and published outcomes as part of the review process. Inadequate registration should be considered a major limitation that introduces a serious risk of bias. Additionally, we encourage increased attention by researchers who conduct trials of MBT to factors in trial design that reduce bias, as well as efforts to conduct trials with adequate sample sizes. Ideally, a smaller number of large, adequately powered trials that provide robust evidence will be conducted going forward rather than a large number of small underpowered trials that tend to fragment the literature, as often occurs presently.

## Supporting Information

S1 Appendix Search Strategies. (DOCX)

S2 Appendix Characteristics of Mindfulness-Based Therapy Studies in Analysis. (DOCX)

S3 Appendix Results from Mindfulness-Based Therapy Studies Included in Analysis. (DOCX)

S4 Appendix Characteristics of Mindfulness-Based Therapy Trial Registrations Included in Analysis. (DOCX)

S5 Appendix Characteristics of Mindfulness-Based Therapy Systematic Reviews and Meta-Analyses Included in Analysis. (DOCX)

S6 Appendix Characteristics of Mindfulness-Based Therapy Systematic Reviews and Meta-Analyses Included in Analysis. (DOCX)

S1 PRISMA Checklist (DOC)
