# Supplementary material for: Reporting of Positive Results in Randomized Controlled Trials of Mindfulness-Based Mental Health Interventions
Source: PLoS One. 2016 Apr 8;11(4):e0153220. doi: 10.1371/journal.pone.0153220 (PMC4825994; doi:10.1371/journal.pone.0153220)
Supplement: S1 Appendix — (DOCX) [file pone.0153220.s001.docx]

**S1 Appendix. Search Strategies**

**Excess Statistical Significance in Published Mindfulness-based Therapy Randomized Controlled Trials**

**CINAHL:** TI (mindful* AND random*) OR AB ( mindful* AND random* ).

**Cochrane CENTRAL:** mindful* AND random* in title abstract

**EMBASE:** (mindful* and random*).ti,ab.

**ISI Web of Knowledge:** Title=(mindful* AND random*) OR Topic=(mindful* AND random*)

**MEDLINE:** (mindful*[Title/Abstract]) AND random*[Title/Abstract].

**PsycInfo:** ((mindful* and random*).ab,ti).

**SCOPUS:** TITLE-ABS-KEY(mindful* AND random*)

**Risk of Publication Bias and Selective Outcome Reporting in MBT Trial Registrations**

Mindfulness

**Assessment of Possible Reporting Biases in Systematic Reviews and Meta-Analyses of MBT**

**CINAHL:** TI (mindfulness AND (systematic review OR meta-analysis)) OR AB (mindfulness AND (systematic review OR meta-analysis)).

**Cochrane CENTRAL:** mindfulness AND (systematic review OR meta-analysis) in title abstract

**EMBASE:** (mindfulness and (systematic review OR meta-analysis)).ti,ab.

**ISI Web of Knowledge:** Title=(mindfulness AND (systematic review OR meta-analysis)) OR Topic=(mindfulness AND (systematic review OR meta-analysis))

**MEDLINE:** (mindfulness[Title/Abstract]) AND (systematic review OR meta-analysis)[Title/Abstract].

**PsycInfo:** ((mindfulness and (systematic review OR meta-analysis)).ab,ti).

**SCOPUS:** TITLE-ABS-KEY(mindfulness AND (systematic review OR meta-analysis)
