# Supplementary material for: Reporting of Positive Results in Randomized Controlled Trials of Mindfulness-Based Mental Health Interventions
Source: PLoS One. 2016 Apr 8;11(4):e0153220. doi: 10.1371/journal.pone.0153220 (PMC4825994; doi:10.1371/journal.pone.0153220)
Supplement: S2 Appendix — (DOCX) [file pone.0153220.s002.docx]

**S2 Appendix. Characteristics of Mindfulness-Based Therapy Studies in Analysis**

| **First Author, Year**  **Journal**^a^ | **Country(ies)** | **Population** | **Clinical or non-clinical population?** | **Did patient eligibility criteria include a minimum threshold for mental health symptoms?** | **Treatment type** |
| --- | --- | --- | --- | --- | --- |
| Alberts, 2012  Appetite | Netherlands | Disordered eating behavior | Clinical | Yes | MBCT |
| Alterman, 2004  J Subst Use | United States | Substance use disorders | Clinical | Yes | Other |
| Anderson, 2007  Clin Psychol | Canada | Non-clinical population | Non-clinical | No | Other |
| Asmaee, 2012  Iran J Public Health | Iran | Generalized anxiety disorder | Clinical | Yes | Other |
| Astin, 1997  Psychother Psychosom | United States | Non-clinical population | Non-clinical | No | Other |
| Azargoon, 2010  J Psychol | Iran | Depression | Clinical | Yes | Other |
| Barnhofer, 2009  Behav Res Ther Crane, 2012  Cogn Ther Res | United Kingdom | Chronic recurrent depression | Clinical | Yes | MBCT |
| Benn, 2012  Dev Psychol | United States | Non-clinical population | Non-clinical | No | Other |
| Berghmans, 2010  J Ther Comport Cognit | France | Non-clinical population | Non-clinical | No | Other |
| Berghmans, 2012  Ann Med Psychol | France | Type 1 diabetes | Clinical | No | Other |
| Biegel, 2009  J Consult Clin Psychol Brown, 2011  Psychol Assess | United States | Outpatient psychiatric facility | Clinical | Yes | Other |
| Bieling, 2012  J Consult Clin Psychol Segal, 2010  Arch Gen Psychiatry | Canada | History of depression | Clinical | Yes | MBCT |
| Bondolfi, 2010  J Affect Disord  Gex-Fabry, 2012  J Psychiatry Res  Jermann, 2013  Cogn Ther Res | Switzerland | Remission from recurrent depression | Clinical | Yes | MBCT |
| Bowen, 2009  Subst Abus  Chawla, 2010  Diss Abs  Witkiewitz, 2010  J Consult Clin Psychol | United States | Substance use disorders | Clinical | Yes | Other |
| Branstrom, 2012  Int J Behav Med Branstrom, 2010  Ann Behav Med  Branstrom, 2013  Psychosomatics | Sweden | Cancer | Clinical | No | Other |
| Brown, 2013  Clin J Pain | United Kingdom | Musculoskeletal pain | Clinical | No | Other |
| Carmody, 2011  Menopause | United States | Non-clinical population | Non-clinical | No | Other |
| Carson, 2004  Behav Ther  Carson, 2003  Diss Abs  Carson, 2007  J Marital Fam Ther | United States | Non-clinical population | Non-clinical | Yes | Other |
| Chadwick, 2009  Behav Cogn Psychother | United Kingdom | Psychosis | Clinical | Yes | Other |
| Chien, 2013  Psychiatr Serv | China | Schizophrenia | Clinical | Yes | Other |
| Chiesa, 2012  J Altern Complement Med | Italy | Major depression | Clinical | Yes | MBCT |
| Chu, 2010  Stress Health | Taiwan | Non-clinical population | Non-clinical | No | Other |
| Clark, 2012  J Evid Based Complement Alternat Med | United States | Cancer | Clinical | No | Other |
| Crane, 2008  Cognit Ther Res Barnhofer, 2007  NeuroReport  Hepburn, 2009  Br J Clin Psychol  Williams, 2008  J Affect Dis | United Kingdom | History of depression and suicidality | Clinical | Yes | MBCT |
| Daubenmier, 2011  J Obes  Daubenmeier, 2012  Psychoneuroendocrinology | United States | Obese or overweight | Clinical | No | Other |
| Davis, 2009  Diss Abs | United States | Overweight | Clinical | No | Other |
| De la Fuente, 2010  Psicotema | Spain | Non-clinical population | Non-clinical | No | Other |
| De Vibe, 2006  Tidsske Nor Laegeforen | Norway | Stress and chronic illness | Clinical | No | Other |
| Duncan, 2012  J Pain Symptom Manage | United States | Human immunodeficiency virus | Clinical | Yes | Other |
| Dziok, 2011  Diss Abs | United States | Non-clinical population | Non-clinical | No | Other |
| Esmer, 2010  J Am Osteopath Assoc | United States | Failed back surgery syndrome | Clinical | No | Other |
| Foley, 2010  J Consult Clin Psychol | Australia | Cancer | Clinical | No | MBCT |
| Frsivold, 2009  Diss Abs | United States | Non-clinical population | Non-clinical | No | Other |
| Gallegos, 2013  Aging Ment Health Moynihan, 2013  Neuropsychobiology | United States | Non-clinical population | Non-clinical | No | Other |
| Gayner, 2012  J Behav Med | Canada | Human immunodeficiency virus | Clinical | No | Other |
| Geschwind, 2012  Br J Psychiatry Geschwind, 2011  J Consult Clin Psychol | Netherlands | History of depression | Clinical | Yes | MBCT |
| Godfrin, 2010  Behav Res Ther | Belgium | History of depression | Clinical | Yes | MBCT |
| Gross, 2010  Altern Ther Health Med Sherr, 2010  Diss Abs | United States | Solid organ transplant recipients | Clinical | No | Other |
| Grossman, 2010  Neurology | Switzerland | Multiple sclerosis | Clinical | No | Other |
| Hartmann, 2012  Diabetes Care | Germany | Type 2 diabetes | Clinical | No | Other |
| Henderson, 2012  Breast Cancer Res Treat  Henderson, 2013  Integr Cancer Ther | United States | Breast cancer | Clinical | No | Other |
| Hoffman, 2012  J Clin Oncol | United Kingdom | Breast cancer | Clinical | No | Other |
| Hoge, 2013  J Clin Psychiatry | United States | Generalized anxiety disorder | Clinical | Yes | Other |
| Jain, 2007  Ann Behav Med | United States | Non-clinical population | Non-clinical | No | Other |
| Jensen, 2012  J Exp Psychol Gen | Denmark | Non-clinical population | Non-clinical | No | Other |
| Johansson, 2012  Brain Inj | Sweden | Stroke or traumatic brain injury | Clinical | No | Other |
| Kang, 2009  Nurse Educ Today | South Korea | Non-clinical population | Non-clinical | No | Other |
| Kaviani, 2012  Arch Psychiatry Psychother | United Kingdom | Subclinical depression | Clinical | Yes | MBCT |
| Kaviani, 2011  Int J Psychol Psychol Ther | United Kingdom | Non-clinical population | Non-clinical | No | MBCT |
| Kearney, 2013  J Clin Psychol | United States | Posttraumatic stress disorder | Clinical | Yes | Other |
| Key, 2011  Diss Abs | Canada | Non-clinical population | Non-clinical | No | Other |
| Kitsumban, 2009  Thai J Nurs Res | Thailand | Depression | Clinical | Yes | Other |
| Klatt, 2009  Health Educ Behav | United States | Non-clinical population | Non-clinical | No | Other |
| Kogler, 2013  Palliat Support Care | Germany | Non-clinical population | Non-clinical | No | Other |
| Kristeller, 2013  Mindfulness | United States | Obese or overweight | Clinical | Yes | Other |
| Kuyken, 2008  J Consult Clin Psych Kuyken, 2010  Behav Res Ther | United Kingdom | Recurrent depression | Clinical | Yes | MBCT |
| Lee, 2011  J Subst Use | Taiwan | Incarcerated substance abusers | Clinical | No | Other |
| Lee, 2007  J Psychosom Res  Kim, 2009  Depress Anxiety | South Korea | Anxiety disorder | Clinical | Yes | Other |
| Lee, 2010  Stress Health | South Korea | Non-clinical population | Non-clinical | No | Other |
| Lengacher, 2009  Psychooncology Lengacher, 2011  J Behav Med  Lengacher, 2012  J Behav Med | United States | Cancer | Clinical | No | Other |
| Lerman, 2012  Ann Surg Oncol | United States | Cancer | Clinical | No | Other |
| Lopez-Rodriguez, 2012  J Res Educ Psychol | Spain | Non-clinical population | Non-clinical | No | Other |
| Ma, 2004  J Consult Clin Psychol | United Kingdom | Recurrent depression | Clinical | Yes | MBCT |
| Mackenzie, 2006  Appl Nurs Res | Canada | Non-clinical population | Non-clinical | No | Other |
| Madani, 2013  Int Med J | Iran | Obsessive-compulsive disorder | Clinical | Yes | Other |
| Malarkey, 2013  Brain Behav Immun | United States | Risk of cardiovascular disease | Clinical | No | Other |
| Manas, 2011  Clin Salud | Spain | Non-clinical population | Non-clinical | No | Other |
| Marfurt, 2006  Diss Abs | United States | Substance use disorders | Clinical | Yes | Other |
| McManus, 2012  J Consult Clin Psychol | United Kingdom | Health anxiety | Clinical | Yes | MBCT |
| Morone, 2009  Pain Med | United States | Chronic low back pain | Clinical | No | Other |
| Murray, 2005  Diss Abs | United States | Non-clinical population | Non-clinical | No | Other |
| Nyklicek, 2012  J Behav Med Nyklicek, 2008  Ann Behav Med | Netherlands | Distress | Clinical | Yes | Other |
| Oken, 2010  J Altern Complement Med | United States | Non-clinical population | Non-clinical | Yes | MBCT |
| Oman, 2008  J Am Coll Health Shapiro, 2008  J Clin Psychol  Shapiro, 2011  J Clin Psychol | United States | Non-clinical population | Non-clinical | No | Other |
| Omidi, 2013  Iran Red Crescent Med J | Iran | Major depressive disorder | Clinical | Yes | MBCT |
| Ortner, 2007  Motiv Emot | Canada | Non-clinical population | Non-clinical | No | Other |
| Parra-Delgado, 2013  Cogn Ther Res | Spain | Fibromyalgia | Clinical | No | MBCT |
| Pbert, 2012  Thorax | United States | Asthma | Clinical | No | Other |
| Perez-Blasco, 2013  Arch Womens Ment Health | Spain | Non-clinical population | Non-clinical | No | Other |
| Perich, 2013  Acta Psychiatr Scand | Australia | Bipolar disorder | Clinical | Yes | MBCT |
| Perkins, 1999  Diss Abs | United States | Inmates | Clinical | No | Other |
| Pinniger, 2012  Complement Ther Med | Australia | Depression | Clinical | No | Other |
| Pinniger, 2013  Am J Dance Res | Australia | Depression | Clinical | No | Other |
| Pipe, 2009  J Nurs Adm | United States | Non-clinical population | Non-clinical | No | Other |
| Plews-Ogan, 2005  J Gen Intern Med | United States | Chronic musculoskeletal pain | Clinical | No | Other |
| Poelke, 2009  Diss Abs | United States | Overweight | Clinical | No | Other |
| Potek, 2012  Diss Abs | United States | Non-clinical population | Non-clinical | No | Other |
| Pradhan, 2007  Arthritis Rheum | United States | Rheumatoid arthritis | Clinical | No | Other |
| Raes, 2013  Mindfulness | Belgium | Non-clinical population | Non-clinical | No | Other |
| Rimes, 2013  Clin Psychol Psychother | United Kingdom | Chronic fatigue syndrome | Clinical | No | MBCT |
| Robins, 2012  J Clin Psychol Keng, 2012  J Cogn Psychother | United States | Non-clinical population | Non-clinical | No | Other |
| Roeser, 2013  J Educ Psychol | Canada/ United States | Non-clinical population | Non-clinical | No | Other |
| Rosdahl, 2003  Diss Abs | United States | Tension headaches | Clinical | No | Other |
| Sagula, 1999  Diss Abs | United States | Chronic pain | Clinical | No | Other |
| Schmidt, 2011  Pain | Germany | Fibromyalgia | Clinical | No | Other |
| Semple, 2010  J Child Fam Studies Lee, 2006  Diss Abs Semple, 2005  Diss Abs | United States | Non-clinical population | Non-clinical | No | MBCT |
| Sephton, 2007  Arthritis Rheum | United States | Fibromyalgia | Clinical | No | Other |
| Seyed-Alinaghi, 2012  Psychosom Med | Iran | Human immunodeficiency virus | Clinical | No | Other |
| Shahar, 2010  Int J Cog Ther Britton, 2006  Diss Abs Britton, 2010  Psychosom Med Britton, 2012  Psychother Psychosom | United States | Recurrent depression | Clinical | Yes | MBCT |
| Shahrestani, 2012  Iranian J Obstet, Gynecol, Infertility | Iran | Infertile women | Clinical | Yes | MBCT |
| Shapiro, 1998  J Behav Med | United States | Non-clinical population | Non-clinical | No | Other |
| Shapiro, 2003  J Psychosom Res  Shapiro, 2002  Diss Abs | United States | Breast cancer | Clinical | No | Other |
| Shapiro, 2005  Int J Stress Manag | United States | Non-clinical population | Non-clinical | No | Other |
| Skovjberg, 2012  Scand J Psychol | Denmark | Multiple chemical sensitivities | Clinical | No | MBCT |
| Speca, 2000  Psychosom Med | Canada | Cancer | Clinical | No | Other |
| Spek, 2013  Res Dev Disabil | Netherlands | Autism spectrum disorder | Clinical | Yes | MBCT |
| Tacon, 2003  Fam Community Health | United States | Heart disease | Clinical | No | Other |
| Tanay, 2012  Behav Ther Jislin-Goldberg, 2012  J Pos Psychol | Israel | Non-clinical population | Non-clinical | No | Other |
| Teasdale, 2000  J Consult Clin Psychol | United Kingdom | Recurrent depression | Clinical | Yes | MBCT |
| Tipsord, 2009  Diss Abs | United States | Non-clinical population | Non-clinical | No | Other |
| Van Aalderen, 2012  Psychol Med  Van der Hurk, 2012  J Exp Psychopathology | Netherlands | History of depression | Clinical | Yes | MBCT |
| Van der Lee, 2012  Psychooncology | Netherlands | Cancer | Clinical | No | MBCT |
| Van Son, 2013  Diabetes Care | Netherlands | Diabetes | Clinical | Yes | MBCT |
| Vieten, 2008  Arch Womens Ment Health | United States | Non-clinical population | Non-clinical | Yes | Other |
| Vollestad, 2011  Behav Res Ther | Norway | Anxiety disorders | Clinical | Yes | Other |
| White, 2012  J Pediatr Health Care White, 2010  Diss Abs | United States | Non-clinical population | Non-clinical | No | Other |
| Williams, 2001  Am J Health Promot | United States | Non-clinical population | Non-clinical | No | Other |
| Wolever, 2012  J Occup Health Psychol | United States | Non-clinical population | Non-clinical | Yes | Other |
| Wong, 2011  Diss Abs | China | Chronic pain | Clinical | No | Other |
| Wong, 2011  Clin J Pain | China | Chronic pain | Clinical | No | Other |
| Wurtzen, 2013  Eur J Cancer | Denmark | Breast cancer | Clinical | No | Other |
| Zangi, 2012  Ann Rheum Dis | Norway | Inflammatory rheumatic joint diseases | Clinical | No | Other |
| Zautra, 2008  J Consult Clin Psychol | United States | Rheumatoid arthritis | Clinical | No | Other |
| Zernicke, 2012  Int J Behav Med | Canada | Irritable bowel syndrome | Clinical | No | Other |

Abbreviations: MBCT= Mindfulness-based cognitive therapy

**^a^** Multiple publications for the same trial were coded as a single trial.
