# Supplementary material for: Reporting of Positive Results in Randomized Controlled Trials of Mindfulness-Based Mental Health Interventions
Source: PLoS One. 2016 Apr 8;11(4):e0153220. doi: 10.1371/journal.pone.0153220 (PMC4825994; doi:10.1371/journal.pone.0153220)
Supplement: S3 Appendix — (DOCX) [file pone.0153220.s003.docx]

**S3 Appendix. Results from Mindfulness-Based Therapy Studies Included in Analysis**

| **First Author, Year**  **Journal**^a^ | **Based on reporting of mental health outcomes in abstract, was study reported positive or negative?** | **For negative studies, was abstract presented with a caveat?** | **For negative studies, were positive within-group outcomes presented?** | **Based on conclusions in study abstract, was study reported positive, negative, or mixed?** | **N analyzed in intervention group/ control group** | **% Power (ES=**  **0.55)** | **Registered Prior to Data Collection** |
| --- | --- | --- | --- | --- | --- | --- | --- |
| Alberts, 2012  Appetite | Positive | NA | NA | Positive | 12/14 | 26.9 | Not registered |
| Alterman, 2004  J Subst Use | Positive | NA | NA | Negative | 15/10 | 25.2 | Not registered |
| Anderson, 2007  Clin Psychol | Positive | NA | NA | Positive | 39/33 | 63.1 | Not registered |
| Asmaee, 2012  Iran J Public Health^bc^ | Positive | NA | NA | Positive | 16/15 | 31.6 | Not registered |
| Astin, 1997  Psychother Psychosom | Positive | NA | NA | Positive | 12/7 | 19.4 | Not registered |
| Azargoon, 2010  J Psychol | Positive | NA | NA | Positive | 18/18 | 36.1 | Not registered |
| Barnhofer, 2009  Behav Res Ther Crane, 2012  Cogn Ther Res | Positive | NA | NA | Positive | 14/14 | 28.9 | Not registered |
| Benn, 2012  Dev Psychol | Positive | NA | NA | Positive | 30/29 | 54.6 | Not registered |
| Berghmans, 2010  J Ther Comport Cognit | Negative | No caveat | Yes | Positive | 10/9 | 20.4 | Not registered |
| Berghmans, 2012  Ann Med Psychol^d^ | Positive | NA | NA | Positive | 10/7 | 18.2 | Not registered |
| Biegel, 2009  J Consult Clin Psychol Brown, 2011  Psychol Assess | Positive | NA | NA | Positive | 50/52 | 78.5 | Not registered |
| Bieling, 2012  J Consult Clin Psychol^d^ Segal, 2010  Arch Gen Psychiatry | Positive | NA | NA | Positive | 26/30 | 52.2 | Prospective registration |
| Bondolfi, 2010  J Affect Disord  Gex-Fabry, 2012  J Psychiatry Res  Jermann, 2013  Cogn Ther Res | Positive | NA | NA | Positive | 27/28 | 51.7 | Not registered |
| Bowen, 2009  Subst Abus^c^  Chawla, 2010  Diss Abs  Witkiewitz, 2010  J Consult Clin Psychol | Positive | NA | NA | Positive | 93/75 | 94.1 | Not registered |
| Branstrom, 2012  Int J Behav Med Branstrom, 2010  Ann Behav Med  Branstrom, 2013  Psychosomatics | Positive | NA | NA | Mixed or inconclusive | 32/39 | 62.3 | Not registered |
| Brown, 2013  Clin J Pain | Positive | NA | NA | Positive | 15/13 | 28.7 | Not registered |
| Carmody, 2011  Menopause | Positive | NA | NA | Positive | 48/44 | 74.1 | Not registered |
| Carson, 2004  Behav Ther  Carson, 2003  Diss Abs  Carson, 2007  J Marital Fam Ther | Positive | NA | NA | Positive | 44/44 | 72.3 | Not registered |
| Chadwick, 2009  Behav Cogn Psychother | Negative | No caveat | Yes | Mixed or inconclusive | 9/9 | 19.5 | Not registered |
| Chien, 2013  Psychiatr Serv | Positive | NA | NA | Positive | 48/48 | 76.0 | Not registered |
| Chiesa, 2012  J Altern Complement Med | Positive | NA | NA | Positive | 9/7 | 17.4 | Not registered |
| Chu, 2010  Stress Health | Positive | NA | NA | Positive | 10/9 | 20.4 | Not registered |
| Clark, 2012  J Evid Based Complement Alternat Med | Negative | Yes, caveat presented | Yes | Positive | 5/7 | 13.6 | Non-prospective registration |
| Crane, 2008  Cognit Ther Res Barnhofer, 2007  NeuroReport  Hepburn, 2009  Br J Clin Psychol  Williams, 2008  J Affect Dis | Positive | NA | NA | Positive | 19/23 | 41.0 | Prospective registration |
| Daubenmier, 2011  J Obes  Daubenmeier, 2012  Psychoneuroendocrinology | Positive | NA | NA | Positive | 19/21 | 39.5 | Prospective registration |
| Davis, 2009  Diss Abs^e^ | Negative | No caveat | Yes | Negative | 20/22 | 41.2 | Not registered |
| De la Fuente, 2010  Psicotema^d^ | Positive | NA | NA | Positive | 24/22 | 44.5 | Not registered |
| De Vibe, 2006  Tidsske Nor Laegeforen | Positive | NA | NA | Positive | 91/42 | 83.3 | Not registered |
| Duncan, 2012  J Pain Symptom Manage | Positive | NA | NA | Positive | 40/36 | 65.7 | Prospective registration |
| Dziok, 2011  Diss Abs | Positive | NA | NA | Positive | 23/6 | 21.2 | Not registered |
| Esmer, 2010  J Am Osteopath Assoc | Positive | NA | NA | Positive | 15/10 | 25.2 | Not registered |
| Foley, 2010  J Consult Clin Psychol | Positive | NA | NA | Positive | 55/60 | 83.2 | Not registered |
| Frsivold, 2009  Diss Abs | Negative | No caveat | Yes | Mixed or inconclusive | 20/18 | 37.8 | Not registered |
| Gallegos, 2013  Aging Ment Health^d^ Moynihan, 2013  Neuropsychobiology | Positive | NA | NA | Positive | 100/100 | 97.2 | Non-prospective registration |
| Gayner, 2012  J Behav Med | Positive | NA | NA | Positive | 78/39 | 79.4 | Non-prospective registration |
| Geschwind, 2012  Br J Psychiatry Geschwind, 2011  J Consult Clin Psychol | Positive | NA | NA | Positive | 64/66 | 87.5 | Prospective registration |
| Godfrin, 2010  Behav Res Ther | Positive | NA | NA | Positive | 52/54 | 80.1 | Prospective registration |
| Gross, 2010  Altern Ther Health Med Sherr, 2010  Diss Abs | Positive | NA | NA | Positive | 63/59 | 85.3 | Prospective registration |
| Grossman, 2010  Neurology | Positive | NA | NA | Positive | 76/74 | 91.7 | Prospective registration |
| Hartmann, 2012  Diabetes Care | Positive | NA | NA | Positive | 53/57 | 81.5 | Not registered |
| Henderson, 2012  Breast Cancer Res Treat  Henderson, 2013  Integr Cancer Ther | Positive | NA | NA | Positive | 53/58 | 81.8 | Not registered |
| Hoffman, 2012  J Clin Oncol | Positive | NA | NA | Positive | 103/111 | 97.9 | Not registered |
| Hoge, 2013  J Clin Psychiatry | Positive | NA | NA | Positive | 48/41 | 72.5 | Prospective registration |
| Jain, 2007  Ann Behav Med | Positive | NA | NA | Positive | 27/30 | 53.1 | Not registered |
| Jensen, 2012  J Exp Psychol Gen^d^ | Positive | NA | NA | Positive | 16/16 | 32.5 | Not registered |
| Johansson, 2012  Brain Inj | Positive | NA | NA | Positive | 12/14 | 26.9 | Not registered |
| Kang, 2009  Nurse Educ Today | Positive | NA | NA | Positive | 16/16 | 32.5 | Not registered |
| Kaviani, 2012  Arch Psychiatry Psychother | Positive | NA | NA | Positive | 14/15 | 29.8 | Not registered |
| Kaviani, 2011  Int J Psychol Psychol Ther | Positive | NA | NA | Positive | 17/19 | 36.0 | Not registered |
| Kearney, 2013  J Clin Psychol | Positive | NA | NA | Mixed or inconclusive | 25/22 | 45.3 | Prospective registration |
| Key, 2011  Diss Abs | Positive | NA | NA | Positive | 31/36 | 59.9 | Not registered |
| Kitsumban, 2009  Thai J Nurs Res | Positive | NA | NA | Positive | 27/27 | 50.9 | Not registered |
| Klatt, 2009  Health Educ Behav^d^ | Positive | NA | NA | Positive | 22/20 | 41.2 | Not registered |
| Kogler, 2013  Palliat Support Care | Positive | NA | NA | Positive | 73/57 | 87.0 | Not registered |
| Kristeller, 2013  Mindfulness | Positive | NA | NA | Positive | 40/35 | 65.0 | Not registered |
| Kuyken, 2008  J Consult Clin Psych Kuyken, 2010  Behav Res Ther | Positive | NA | NA | Positive | 61/62 | 85.7 | Prospective registration |
| Lee, 2011  J Subst Use | Positive | NA | NA | Positive | 10/14 | 24.6 | Not registered |
| Lee, 2007  J Psychosom Res  Kim, 2009  Depress Anxiety | Positive | NA | NA | Positive | 21/20 | 40.4 | Not registered |
| Lee, 2010  Stress Health | Positive | NA | NA | Positive | 30/30 | 55.4 | Not registered |
| Lengacher, 2009  Psychooncology Lengacher, 2011  J Behav Med  Lengacher, 2012  J Behav Med | Positive | NA | NA | Positive | 40/42 | 69.1 | Prospective registration |
| Lerman, 2012  Ann Surg Oncol^d^ | Positive | NA | NA | Positive | 48/20 | 53.1 | Not registered |
| Lopez-Rodriguez, 2012  J Res Educ Psychol^d^ | Positive | NA | NA | Positive | 23/23 | 44.6 | Not registered |
| Ma, 2004  J Consult Clin Psychol^d^ | Positive | NA | NA | Positive | 36/37 | 64.0 | Not registered |
| Mackenzie, 2006  Appl Nurs Res | Positive | NA | NA | Positive | 16/14 | 30.6 | Not registered |
| Madani, 2013  Int Med J | Positive | NA | NA | Positive | 12/12 | 25.2 | Not registered |
| Malarkey, 2013  Brain Behav Immun | Negative | Yes, caveat presented | No | Mixed or inconclusive | 84/86 | 94.6 | Not registered |
| Manas, 2011  Clin Salud | Positive | NA | NA | Positive | 16/15 | 31.6 | Not registered |
| Marfurt, 2006  Diss Abs | Negative | No caveat | No | Negative | 5/5 | 12.0 | Not registered |
| McManus, 2012  J Consult Clin Psychol | Positive | NA | NA | Positive | 36/38 | 64.6 | Not registered |
| Morone, 2009  Pain Med | Negative | No caveat | Yes | Positive | 16/19 | 35.0 | Prospective registration |
| Murray, 2005  Diss Abs | Negative | No caveat | No | Mixed or inconclusive | 11/11 | 23.3 | Not registered |
| Nyklicek, 2012  J Behav Med Nyklicek, 2008  Ann Behav Med | Positive | NA | NA | Positive | 72/74 | 91.0 | Not registered |
| Oken, 2010  J Altern Complement Med | Positive | NA | NA | Positive | 8/9 | 18.5 | Not registered |
| Oman, 2008  J Am Coll Health Shapiro, 2008  J Clin Psychol  Shapiro, 2011  J Clin Psychol | Positive | NA | NA | Positive | 15/15 | 30.7 | Not registered |
| Omidi, 2013  Iran Red Crescent Med J | Positive | NA | NA | Positive | 30/30 | 55.4 | Not registered |
| Ortner, 2007  Motiv Emot^d^ | Positive | NA | NA | Positive | 21/24 | 43.6 | Not registered |
| Parra-Delgado, 2013  Cogn Ther Res | Positive | NA | NA | Positive | 15/16 | 31.6 | Not registered |
| Pbert, 2012  Thorax | Positive | NA | NA | Positive | 42/41 | 69.7 | Prospective registration |
| Perez-Blasco, 2013  Arch Womens Ment Health | Positive | NA | NA | Positive | 13/8 | 21.4 | Not registered |
| Perich, 2013  Acta Psychiatr Scand | Positive | NA | NA | Mixed or inconclusive | 48/47 | 75.6 | Non-prospective registration |
| Perkins, 1999  Diss Abs | Positive | NA | NA | Positive | 49/48 | 76.5 | Not registered |
| Pinniger, 2012  Complement Ther Med | Positive | NA | NA | Positive | 16/29 | 40.8 | Not registered |
| Pinniger, 2013  Am J Dance Res | Positive | NA | NA | Positive | 11/23 | 30.7 | Not registered |
| Pipe, 2009  J Nurs Adm | Positive | NA | NA | Positive | 15/17 | 32.4 | Not registered |
| Plews-Ogan, 2005  J Gen Intern Med | Positive | NA | NA | Positive | 5/8 | 14.3 | Not registered |
| Poelke, 2009  Diss Abs | Positive | NA | NA | Positive | 19/20 | 38.7 | Not registered |
| Potek, 2012  Diss Abs | Positive | NA | NA | Positive | 16/14 | 30.6 | Not registered |
| Pradhan, 2007  Arthritis Rheum | Positive | NA | NA | Mixed or inconclusive | 28/32 | 55.2 | Prospective registration |
| Raes, 2013  Mindfulness | Positive | NA | NA | Positive | 182/175 | 99.9 | Not registered |
| Rimes, 2013  Clin Psychol Psychother | Positive | NA | NA | Positive | 16/19 | 35.0 | Not registered |
| Robins, 2012  J Clin Psychol Keng, 2012  J Cogn Psychother | Positive | NA | NA | Positive | 20/21 | 40.4 | Not registered |
| Roeser, 2013  J Educ Psychol | Positive | NA | NA | Positive | 54/59 | 82.5 | Not registered |
| Rosdahl, 2003  Diss Abs | Negative | No caveat | No | Negative | 34/30 | 58.0 | Not registered |
| Sagula, 1999  Diss Abs^f^ | Positive | NA | NA | Positive | 22/18 | 39.2 | Not registered |
| Schmidt, 2011  Pain | Negative | Yes, caveat is presented | Yes | Mixed or inconclusive | 53/59 | 82.1 | Prospective registration |
| Semple, 2010  J Child Fam Studies Lee, 2006  Diss Abs Semple, 2005  Diss Abs | Negative | Yes, caveat is presented | Yes | Positive | 13/12 | 26.1 | Not registered |
| Sephton, 2007  Arthritis Rheum | Positive | NA | NA | Positive | 51/39 | 72.5 | Not registered |
| Seyed-Alinaghi, 2012  Psychosom Med | Negative | No caveat | Yes | Positive | 87/86 | 94.9 | Non-prospective registration |
| Shahar, 2010  Int J Cog Ther Britton, 2006  Diss Abs Britton, 2010  Psychosom Med Britton, 2012  Psychother Psychosom | Positive | NA | NA | Positive | 26/19 | 42.9 | Not registered |
| Shahrestani, 2012  Iranian J Obstet, Gynecol, Infertility | Positive | NA | NA | Positive | 12/12 | 25.2 | Not registered |
| Shapiro, 1998  J Behav Med | Positive | NA | NA | Positive | 36/37 | 64.0 | Not registered |
| Shapiro, 2003  J Psychosom Res^d^  Shapiro, 2002  Diss Abs | Positive | NA | NA | Positive | 26/28 | 50.9 | Not registered |
| Shapiro, 2005  Int J Stress Manag | Positive | NA | NA | Positive | 10/18 | 26.9 | Not registered |
| Skovjberg, 2012  Scand J Psychol | Negative | Yes, caveat presented | No | Mixed or inconclusive | 12/17 | 29.1 | Prospective registration |
| Speca, 2000  Psychosom Med | Positive | NA | NA | Positive | 53/37 | 71.9 | Not registered |
| Spek, 2013  Res Dev Disabil | Positive | NA | NA | Positive | 20/21 | 40.4 | Not registered |
| Tacon, 2003  Fam Community Health | Positive | NA | NA | Positive | 9/9 | 19.5 | Not registered |
| Tanay, 2012  Behav Ther^b^ Jislin-Goldberg, 2012  J Pos Psychol | Negative | No caveat | Yes | Positive | 17/34 | 44.3 | Not registered |
| Teasdale, 2000  J Consult Clin Psychol^d^ | Positive | NA | NA | Positive | 76/69 | 90.7 | Not registered |
| Tipsord, 2009  Diss Abs | Positive | NA | NA | Positive | 24/26 | 47.8 | Not registered |
| Van Aalderen, 2012  Psychol Med  Van der Hurk, 2012  J Exp Psychopathology | Positive | NA | NA | Positive | 102/103 | 97.5 | Prospective registration |
| Van der Lee, 2012  Psychooncology | Positive | NA | NA | Positive | 59/24 | 61.2 | Not registered |
| Van Son, 2013  Diabetes Care | Positive | NA | NA | Positive | 70/69 | 89.6 | Prospective registration |
| Vieten, 2008  Arch Womens Ment Health | Positive | NA | NA | Positive | 13/18 | 30.9 | Not registered |
| Vollestad, 2011  Behav Res Ther | Positive | NA | NA | Positive | 39/37 | 65.7 | Not registered |
| White, 2012  J Pediatr Health Care White, 2010  Diss Abs | Positive | NA | NA | Positive | 70/85 | 92.3 | Not registered |
| Williams, 2001  Am J Health Promot | Positive | NA | NA | Positive | 32/26 | 53.5 | Not registered |
| Wolever, 2012  J Occup Health Psychol | Positive | NA | NA | Positive | 32/47 | 65.9 | Not registered |
| Wong, 2011  Diss Abs | Positive | NA | NA | Positive | 38/27 | 57.6 | Not registered |
| Wong, 2011  Clin J Pain | Negative | No caveat | Yes | Mixed or inconclusive | 51/48 | 77.3 | Prospective registration |
| Wurtzen, 2013  Eur J Cancer | Positive | NA | NA | Positive | 139/143 | 99.6 | Prospective registration |
| Zangi, 2012  Ann Rheum Dis | Positive | NA | NA | Positive | 34/34 | 60.8 | Not registered |
| Zautra, 2008  J Consult Clin Psychol^d^ | Positive | NA | NA | Positive | 44/43 | 71.8 | Not registered |
| Zernicke, 2012  Int J Behav Med^d^ | Positive | NA | NA | Positive | 43/47 | 73.2 | Not registered |

Abbreviations: N= Number; ES = Effect size; NA= Not applicable

**^a^**Multiple publications for the same trial were coded as a single trial. If there were discrepancies in group sizes between publications, the largest intervention and control group sizes were used. **^b^**Authors indicated that they used an inactive control group, but did not specify the nature of the inactive control. **^c^**Number of patients analyzed could not be extracted, so we recorded the sample size randomized. **^d^**Publication did not report any between-group outcomes, but did report positive within-group mental health outcomes. **^e^**Intervention group consisted of a diet-exercise intervention group with separate, supplementary MBT component (15-30 minute sessions) while control group only had the diet-exercise intervention. The control group was thus categorized as treatment as usual. **^f^**Publication used two MBT intervention groups, which were similar in every way except the duration of sessions. One group met for 45 minutes weekly and another group met for 20 minutes weekly. We used the intervention group with the larger sample size, which in this case, was the 45-minute session intervention group.
