# Supplementary material for: Reporting of Positive Results in Randomized Controlled Trials of Mindfulness-Based Mental Health Interventions
Source: PLoS One. 2016 Apr 8;11(4):e0153220. doi: 10.1371/journal.pone.0153220 (PMC4825994; doi:10.1371/journal.pone.0153220)
Supplement: S4 Appendix — (DOCX) [file pone.0153220.s004.docx]

**S4 Appendix. Characteristics of Mindfulness-Based Therapy Trial Registrations Included in Analysis**

| **Primary contact** | **Trial registry number** | **Trial status listed in registry as of July 04, 2013** | **Population studied** | **Published within 30 months of trial completion?**^a^ | **Classification of published RCT** | **Was trial registered adequately?** |
| --- | --- | --- | --- | --- | --- | --- |
| Berman | NCT00071292 | Completed | Rheumatoid arthritis | No^b,1^ | Positive | No, multiple primary outcomes |
| Bremner | NCT01058031 | Completed | Post-traumatic stress disorder | No | NA | No, metric not specified |
| Chychula | NCT00375531 | Withdrawn | War veterans | No | NA | No, multiple primary outcomes |
| De Klerk | NCT00694668 | Unknown | Adolescents and young adults with deliberate self harm | No | NA | No, multiple primary outcomes |
| Fortin | ISRCTN03419655 | Completed | Systemic lupus erythematosus | No | NA | No, multiple primary outcomes |
| Gross | NCT00367809 | Completed | Solid organ transplant recipients | Yes^2^ | Positive | No, multiple primary outcomes |
| Grossman | NCT00106275 | Unknown | Fibromyalgia | No^b,c,3^ | Negative | No, multiple primary outcomes |
| Grossman | ISRCTN21643919 | Completed | Multiple sclerosis | Yes^4^ | Positive | No, multiple primary outcomes |
| Johnson | NCT00312936 | Completed | Human immunodeficiency virus | No^b,5^ | Positive | No, multiple primary outcomes |
| Kearney | NCT00880152 | Completed | Post-traumatic stress disorder | No^b,6^ | Positive | No, measure not specified |
| Kuyken | ISRCTN12720810 | Completed | Depression | Yes^7^ | Positive | No, timeframe not specified |
| Meadows | ACTRN12605000761662 | Completed | Depression | No | NA | No, multiple primary outcomes |
| Moix | NCT00360802 | Completed | Chronic low back pain | No^d^ | NA | No, multiple primary outcomes |
| Quillian-Wolever | NCT00032760 | Completed | Binge eating disorder | No | NA | No, multiple primary outcomes |
| Segal | NCT00183560 | Completed | Depression | Yes^8,9^ | Positive | No, metric not specified |
| Sheps | NCT00224835 | Unknown | Myocardial ischemia | No | NA | No, multiple primary outcomes |
| Speckens | NCT01038765 | Unknown | Psychiatric | Yes^c,10^ | Positive | No, measure not specified |
| Van Heeringen | NCT00259506 | Completed | Depression | Yes^11^ | Positive | No, multiple primary outcomes |
| Whitman Davis | NCT00936351 | Completed | Schizophrenia | No | NA | No, multiple primary outcomes |
| Williams | ISRCTN55225822 | Completed | Suicidality | Yes^12,13,14^ | Positive | No, multiple primary outcomes |
| Würtzen | NCT00990977 | Completed | Breast cancer | Yes^15^ | Positive | No, multiple primary outcomes |

Abbreviations: RCT= Randomized controlled trial

^a^ Corresponding publications are listed below. ^b^ Trial results were published, but not within 30 months of trial completion. ^c^ Trial, according to the trial registry, had a status of “unknown” because the information in the trial registration had not been verified recently. However, at the time of our search, there existed a publication of trial results linked to the corresponding trial registration number. ^d^ There was a publication linked to this trial registration number with results for a single-arm trial, but the publication was not a report of the randomized controlled trial outcomes described in the trial registration.

**S4 Appendix (continued). Publications Associated with Trial Registrations**

1. Pradhan EK, Baumgarten M, Langenberg P, Handwerger B, Gilpin AK, Magyari T, et al. Effect of mindfulness-based stress reduction in rheumatoid arthritis patients. Arthritis Rheum. 2007;57: 1134-1142.

2. Gross CR, Kreitzer MJ, Thomas W, Reilly-Spong M, Cramer-Bornemann M, Nyman JA, et al. Mindfulness-based stress reduction for solid organ transplant recipients: a randomized controlled trial. Altern Ther Health Med. 2010;16: 30-38.

3. Schmidt S, Grossman P, Schwarzer B, Jena S, Naumann J, Walach H. Treating fibromyalgia with mindfulness-based stress reduction: results from a 3-armed randomized controlled trial. Pain. 2011;152: 361-369.

4. Grossman P, Kappos L, Gensicke H, D’Souza M, Mohr DC, Penner IK, et al. MS quality of life, depression, and fatigue improve after mindfulness training: a randomized trial. Neurology. 2010;75: 1141-1149.

5. Duncan LG, Moskowitz JT, Neilands TB, Dilworth SE, Hacht FM, Johnson MO. Mindfulness-based stress reduction for HIV treatment side effects: a randomized, wait-list controlled trial. J Pain Symptom Manage. 2012;43: 161-171.

6. Kearney DJ, McDermott K, Malte C, Martinez M, Simpson TL. Effects of participation in a mindfulness program for veterans with posttraumatic stress disorder: a randomized controlled pilot study. J Clin Psychol. 2013;69: 14-27.

7. Kuyken W, Byford S, Taylor RS, Watkins E, Holden E, White K. Mindfulness-based cognitive therapy to prevent relapse in recurrent depression. J Consult Clin Psychol. 2008;76: 966-978.

8. Segal ZV, Bieling P, Young T, MacQueen G, Cooke R, Martin L, et al. Antidepressant monotherapy vs sequential pharmacotherapy and mindfulness-based cognitive therapy, or placebo, for relapse prophylaxis in recurrent depression. Arch Gen Psychiatry. 2010;67: 1256-1264.

9. Bieling PJ, Hawley LL, Bloch RT, Corcoran KM, Levitan RD, Young LT, et al. Treatment-specific changes in decentering following mindfulness-based cognitive therapy versus antidepressant medication or placebo for prevention of depressive relapse. J Consult Clin Psychol. 2012;80: 365-372.

10. van Aalderen JR, Donders AR, Giommi F, Spinhoven P, Barendregt HP, Speckens AE. The efficacy of mindfulness-based cognitive therapy in recurrent depressed patients with and without a current depressive episode: a randomized controlled trial. Psychol Med. 2012;42: 989-1001.

11. Godfrin KA, van Heeringen C. The effects of mindfulness-based cognitive therapy on recurrence of depressive episodes, mental health and quality of life: a randomized controlled study. Behav Res Ther. 2010;48: 738-746.

12. Williams JM, Alatiq Y, Crane C, Barnhofer T, Fennell MJ, Duggan DS, et al. Mindfulness-based cognitive therapy (MBCT) in bipolar disorder: preliminary evaluation of immediate effects on between-episode functioning. J Affect Disord. 2008;107: 275-279.

13. Crane C, Barnhofer T, Duggan DS, Hepburn S, Fennell MV, Williams JM. Mindfulness-based cognitive therapy and self-discrepancy in recovered depressed patients with a history of depression and suicidality. Cogn Ther Res. 2008;32: 775-787.

14. Hepburn SR, Crane C, Barnhofer T, Duggan DS, Fennel MJ, Williams JM. Mindfulness-based cognitive therapy may reduce thought suppression in previously suicidal participants: findings from a preliminary study. Br J Clin Psychol. 2009;48: 209-215.

15. Wurtzen H, Dalton SO, Elsass P, Sumbundu AD, Steding-Jensen M, Karlsen RV, et al. Mindfulness significantly reduces self-reported levels of anxiety and depression: results of a randomised controlled trial among 336 Danish women treated for stage I-III breast cancer. Eur J Cancer. 2013;49: 1365-1373.
