# Supplementary material for: Reporting of Positive Results in Randomized Controlled Trials of Mindfulness-Based Mental Health Interventions
Source: PLoS One. 2016 Apr 8;11(4):e0153220. doi: 10.1371/journal.pone.0153220 (PMC4825994; doi:10.1371/journal.pone.0153220)
Supplement: S5 Appendix — (DOCX) [file pone.0153220.s005.docx]

**S5 Appendix. Characteristics of Mindfulness-Based Therapy Systematic Reviews and Meta-Analyses Included in Analysis**

| **First Author,**  **Year, Journal** | **Country** | **Number of MBT RCTs included** | **Number of MBT non-randomized trials included** | **Did authors of the review conduct a statistical test (e.g., asymmetry, fail-safe N) or a visual test (e.g., funnel plot) to assess possible reporting bias?** | **If the authors conducted a statistical or visual test to assess possible reporting bias, what did they conclude?** | **Did the authors of the review comment on reporting bias?** | **If the authors commented on bias, what did they say?** |
| --- | --- | --- | --- | --- | --- | --- | --- |
| Casellas-Grau, 2013, Psychooncology | Spain | 1 | 6 | No | NA | No | NA |
| Chapman, 2013, Mindfulness | United Kingdom | 0 | 11 | No | NA | No | NA |
| Chen, 2012, Depress Anxiety | United States | 14 | 0 | Yes | No publication bias | No | NA |
| Chiesa, 2011, Psychiatry Res | Italy | 12 | 4 | Yes | No publication bias | No | NA |
| Chiesa, 2011, J Altern Complement Med | Italy | 6 | 4 | No | NA | No | NA |
| Chiesa, 2013, Subst Use Misuse | Italy | 14 | 10 | No | NA | No | NA |
| Cramer, 2012, BMC Complement Altern Med | Germany | 3 | 0 | No | NA | No | NA |
| Cramer, 2012, Current Oncol | Germany | 3 | 0 | Yes | Inconclusive | Yes | Bias could not be ruled out due to small number of studies included |
| D’Silva, 2012, Psychosomatics | United States | 11 | 0 | No | NA | No | NA |
| Eberth, 2012, Mindfulness | Germany | 12 | 5 | Yes | Possible publication bias | Yes | Possible publication bias, but if it exists it is small and does not invalidate findings |
| Escuriex, 2011, Mindfulness | United States | 3 | 17 | No | NA | No | NA |
| Fjorback, 2012, Dan Med J | Denmark | 3 | 0 | No | NA | No | NA |
| Fjorback, 2011, Acta Psychiatr Scand | Denmark | 21 | 0 | No | NA | Yes | Publication bias cannot be ruled out due to generally positive findings |
| Galante, 2013, J Res Nurs | United Kingdom | 11 | 0 | Yes | No publication bias | No | NA |
| Hwang, 2012, J Child Fam Stud | Australia | 0 | 7 | No | NA | No | NA |
| Khoury, 2013, Clin Psychol Rev | Canada | 135 | 74 | Yes | No publication bias | No | NA |
| Khoury, Schizophr Res^a^ | Canada | 3 | 10 | Yes | Possible publication bias | No | NA |
| Klainin-Yobas, 2012, Int J Nurs Stud | Singapore | 14 | 25 | Yes | No publication bias | Yes | Strength of meta-analysis was testing for publication bias |
| Langhorst, 2012, Schwerz | Germany | 4 | 4 | No | NA | No | NA |
| Lawrence, 2013, Int J Stroke | United Kingdom | 1 | 3 | No | NA | No | NA |
| Luberto, 2013, Curr Psychiatry Rep^b^ | United States | 17 | 0 | No | NA | No | NA |
| Masuda, 2013, Neuropsychiatry | United States | 4 | 5 | No | NA | No | NA |
| McCarney, 2013, Eur J Psychother Couns | United Kingdom | 2 | 9 | Yes | No publication bias | Yes | No publication bias based on analyses |
| Miro, 2011, Revista De Psicopatología y Psicología Clínica | Spain | 6 | 5 | No | NA | No | NA |
| Musial, 2012, Forsch Komplementmed | Norway | 5 | 14 | No | NA | No | NA |
| Niazi, 2011, N Am J Med Science^b^ | Pakistan | 18 | 0 | No | NA | No | NA |
| Piet, 2011, Clin Psychol Rev | Denmark | 6 | 0 | Yes | No publication bias | No | NA |
| Piet, 2012, J Consult Clin Psychol | Denmark | 9 | 13 | Yes | No publication bias | Yes | Strength of study was testing for publication bias |
| Regehr, 2012, J Affect Disord | Canada | 5 | 4 | No | NA | No | NA |
| Sarris, 2012, J Affect Disord | Australia | 1 | 0 | No | NA | No | NA |
| Sarris, 2012, Evid Based Complement Alternat Med | Australia | 3 | 11 | No | NA | No | NA |
| Senders, 2012, Autoimmune Dis | United States | 1 | 1 | No | NA | No | NA |
| Shennan, 2011, Psychooncology | United Kingdom | 3 | 10 | No | NA | No | NA |
| Shiralkar, 2013, Acad Psychiatry | United States | 1 | 1 | No | NA | No | NA |
| Shonin, 2013, Aggress Violent Behav | United Kingdom | 1 | 1 | No | NA | No | NA |
| Veehof, 2011, Pain | Netherlands | 7 | 8 | Yes | Possible publication bias | Yes | For 1 of 2 outcomes, indicated that funnel plot asymmetry suggests the possibility of publication bias |
| Vollestad, 2012, Br J Clin Psychol | Norway | 4 | 11 | Yes | No publication bias | Yes | No publication bias based on analyses |
| Zainal, 2012, Pyschooncology | Malaysia | 2 | 7 | Yes | Publication bias in opposite direction | Yes | Publication bias in opposite direction, based on trim-and-fill |

Abbreviations: MBT= Mindfulness-based therapies; RCTs= Randomized controlled trials; N= Number; NA= Not applicable

^a^ This review classified controlled trials into waitlist-controlled trials and treatment-controlled trials. Since the method used to classify studies into these two categories differed from the methods used in the present study, all controlled studies were considered randomized controlled trials, regardless of the type of control group. ^b^ Review did not specify whether mindfulness-based therapies reviewed were randomized controlled trials or not.
